# Personalized porous tantalum implants crafted via 3D printing: new horizons in complex cervical-thoracic spinal fusion

**DOI:** 10.3389/fbioe.2025.1625650

**Published:** 2025-08-21

**Authors:** Chang Chen, Huaquan Fan, Ge Chen, Zhong Li, Puquan Wang, Fuyou Wang

**Affiliations:** ^1^ Department of Traditional Chinese Medicine Rehabilitation, Jiangbei Branch of The First Hospital Affiliated to Army Medical University (Third Military Medical University), Chongqing, China; ^2^ Center for Joint Surgery, The First Hospital Affiliated to Army Medical University, Chongqing, China; ^3^ Department of Orthopaedics, Affiliated Hospital of Southwest Medical University, Luzhou, China

**Keywords:** porous tantalum, cervical vertebral deformity, thoracic spine tumor, additive manufacturing, interbody fusion, clinical efficacy

## Abstract

**Background:**

Complex interbody fusion remains challenging, while traditional surgical instruments are not suitable for complex spinal deformities. Porous tantalum (Ta) has excellent osteogenic properties, but there is currently a lack of research on its application in cervical thoracic interbody fusion.

**Objective:**

To introduce the application of selective electron beam melting (SEBM) 3D printing technology in customized porous Ta vertebral fusion implants and evaluate its mid-term clinical efficacy in complex cervical thoracic fusion surgery. Method: Porous Ta implants were manufactured using SEBM technology. The mechanical properties were optimized and characterized. Three patients who underwent complex cervical and thoracic fusion surgery were prospectively recruited. 3D printing technology is used for preoperative planning and customized implant design. Surgical techniques and postoperative management follow standard procedures, with regular follow-up including clinical and imaging evaluations.

**Result:**

Porous Ta implants have satisfactory pore structure and surface characteristics, with mechanical properties. All three surgeries were successful. The operation time is 188–525 min (average 387.7 min), the intraoperative blood loss is 300–1,000 mL (average 695 mL), and the hospitalization time is 21–36 days (average 30.0 days). After an average follow-up of 24.3 months, the patient’s pain symptoms improved significantly and no serious complications occurred.

**Conclusion:**

The use of 3D printed personalized porous tantalum implants in complex spinal fusion procedures is feasible and has shown significant benefits. Future research should focus on validating these results through larger cohorts and long-term follow-up to explore the broader application prospects.

## 1 Introduction

Interbody fusion, widely applied in the treatment of spinal diseases such as lumbar disc herniation, degeneration, tumors and spondylolisthesis, is one of the core surgical procedures for reconstructing spinal stability (Kgomotso et al., 2023) (Försth et al., 2016), thereby alleviating pain and restoring spinal function (He et al., 2023). However, for patients with congenital or traumatic anatomical deformities, traditional surgical instruments may not perfectly match the complex anatomical structures (Ye et al., 2023). The current application of 3D printing technology in orthopedics has became extensive, since it can accurately manufacture implants that fully conform to the patient’s anatomy based on individual imaging data (Mayfield et al., 2022). In complex knee and hip arthroplasty, 3D printed prostheses can better match the joint surface, improve stability and biocompatibility after implantation, thus reducing the incidence of postoperative complications (Periferakis et al., 2024). For complex spinal and pelvic fracture surgery, preoperative models created by 3D printing modeling can intuitively understand the anatomical structure, developing more accurate surgical plans (Hajnal et al., 2025) (Wang et al., 2020). Meanwhile, personalized surgical guiding plates fabricated by 3D printing technology could providing precise guidance for surgical operations (Jha et al., 2022). For complex spinal bone defects, personalized surgical planning and customized implant can effectively reduce surgical trauma and blood loss, shorten surgical time, and thus reduce the incidence of complications (Abel et al., 2023) (Sheha et al., 2019).

Synthetic materials such as titanium (Ti) alloy and polyetheretherketone (PEEK) have some obvious limitations in orthopedic applications (Deng et al., 2022). Ti alloy, despite its high strength, has a much higher elastic modulus than natural human bone, which can easily lead to stress shielding effect after implantation (Johnson et al., 2023). Therefore, metals including Ti may reduce the stress borne by the bone, enhancing osteoclast activity and inhibiting osteoblast activity, and eventually resulting in bone resorption and implant loosening (Segi et al., 2023). While boasting good biocompatibility, radiolucency and chemical stability (Kamenova et al., 2023), PEEK exhibits low osteogenic property and unsatisfactory osseointegration efficiency (Du et al., 2023). Due to the nonporous structure and limited surface area for bone attachment, the surface of PEEK is prone to being encapsulated by fibrous tissue, resulting in insufficient bonding strength at the bone-material interface and an increased risk of fusion failure (Deng et al., 2023) (Sinclair et al., 2012).

Porous tantalum (TM) is currently one of the most popular high-porosity metallic materials, renowned for its excellent bone ingrowth and osteogenesis properties (Ferraro et al., 2023). The 3D-printed porous tantalum interbody fusion implant can closely approximate the modulus of cancellous bone (3–4 GPa), thereby mitigating issues such as stress shielding (Fiani et al., 2021). Porous Ta has been proved to have superior tissue affinity with outstanding osteoconductivity and osteoinductivity, leading to a better bone in-growth (Lebhar et al., 2020). Meanwhile, porous Ta boasts high toughness, malleability, and exceptional fatigue resistance, making it a suitable option for use in orthopedic implants (Jordan et al., 2021). However, the most widely employed method to fabricating TM - chemical vapor deposition (CVD) technique - is complex and costly (Lei et al., 2022). Currently, the development of 3D printing (3DP) technology make it possible to precisely control the porosity, pore size, pore structure, and overall shape of porous tantalum implants, enabling them to better mimic the structure and function of human bone.

Owing to more complex anatomic, proximity to vital organs, and higher technical demands compared to lumbar spine surgery (Liang et al., 2021), surgical procedures involving the thoracic and cervical spine necessitate a greater emphasis on personalized planning to optimize surgical strategies and enhance safety (Tan et al., 2018). However, there is currently a lack of clinical research on the application of 3D-printed porous Ta in interbody fusion, with even fewer studies specifically focusing on its clinical use in cervical and thoracic fusion. This study introduces the application of Selective Electron Beam Melting (SEBM) 3D printing technology to customize porous Ta vertebral body fusion implants, while evaluates the mid-term efficacy of customized porous Ta cage in clinical applications of three case series. This study aims to offer a more ideal option for complex cervical and thoracic vertebral fusion surgeries, while systematically elaborate on the clinical application and mid-term outcomes of 3D printing porous Ta implants in vertebral fusion surgery through three complex spinal fusion cases.

## 2 Methods

### 2.1 General setting in the three-dimensional printing

#### 2.1.1 Parametric calculation of porous structures

**FIGURE 1:**
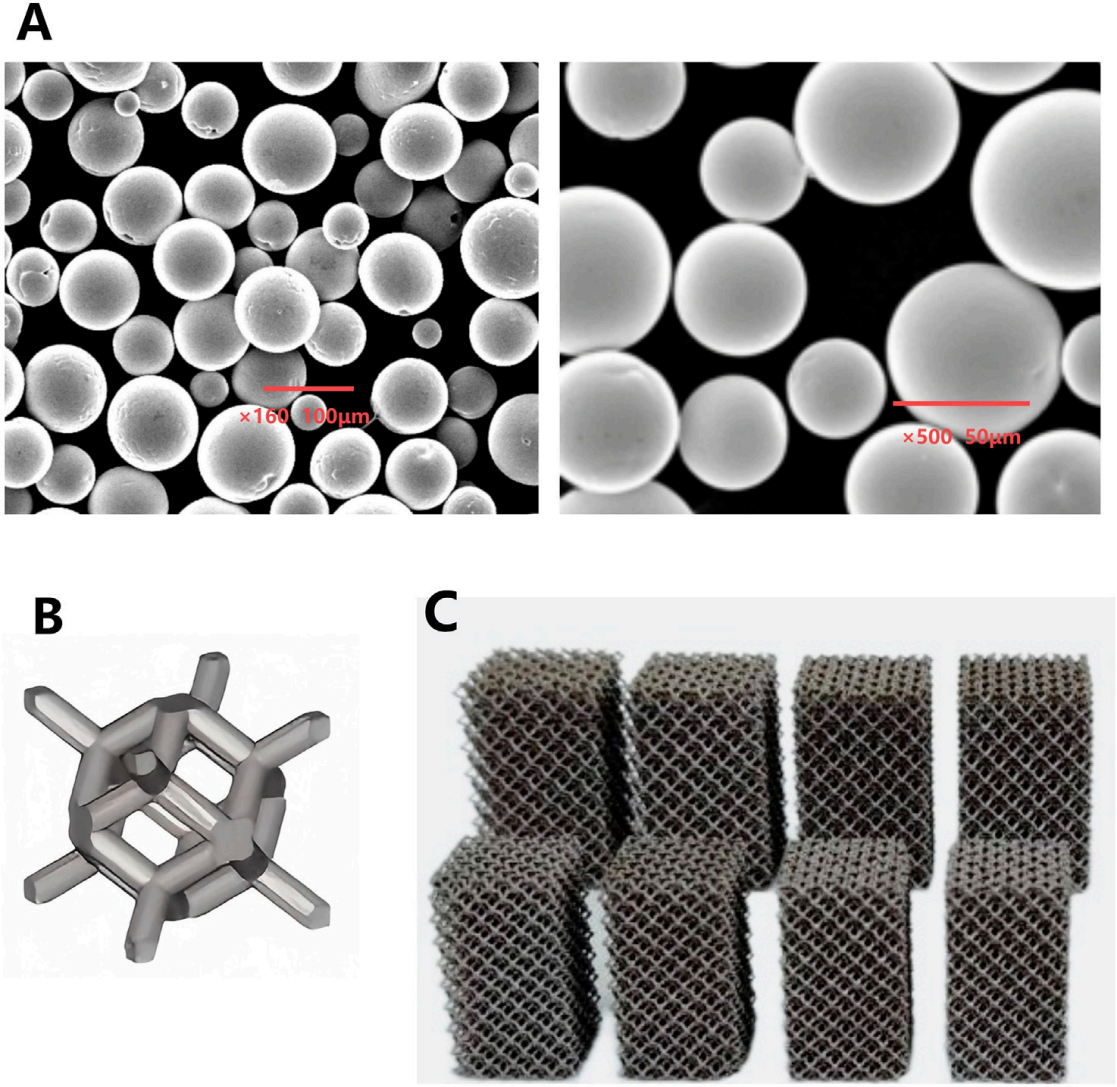
**(A)** Microscopic morphology of Ta powder produced by PREP; **(B)** Schematic illustration of a porous Ta rhombic dodecahedron; **(C)** Porous Ta block specimens with different porosity.

The rhombic dodecahedral cell is composed of 12 congruent rhombus, which has 24 edges and 14 vertices. The acute angle in the rhombus is 70.5° and the obtuse angle is 109.5°, as shown in Figure 1B. Therefore, the relative density of the diamond dodecahedron can be calculated as:

$$\frac{3\sqrt{3}}{2}\pi {\left(\frac{r}{a}\right)}^{2}-\frac{27\sqrt{2}}{4}{\left(\frac{r}{a}\right)}^{3}$$ (1)

Where *r* is the radius of hole edge and *a* is the length of hole edge. It can be seen from Formula 1 that changing the length and diameter of the hole edge can accurately adjust the porosity of the sample, so this formula will be one of the basis for the structural design of personalized porous tantalum implants.

In order to clarify the relationship between these parameters more clearly, the relationship between the aperture of the diamond dodecahedron and the size of the unit cell is analyzed, and the following relationship can be obtained by combining Formula 1:

H=0.816×M−D (2)

M=2.31×a (3)

**FIGURE 2:**
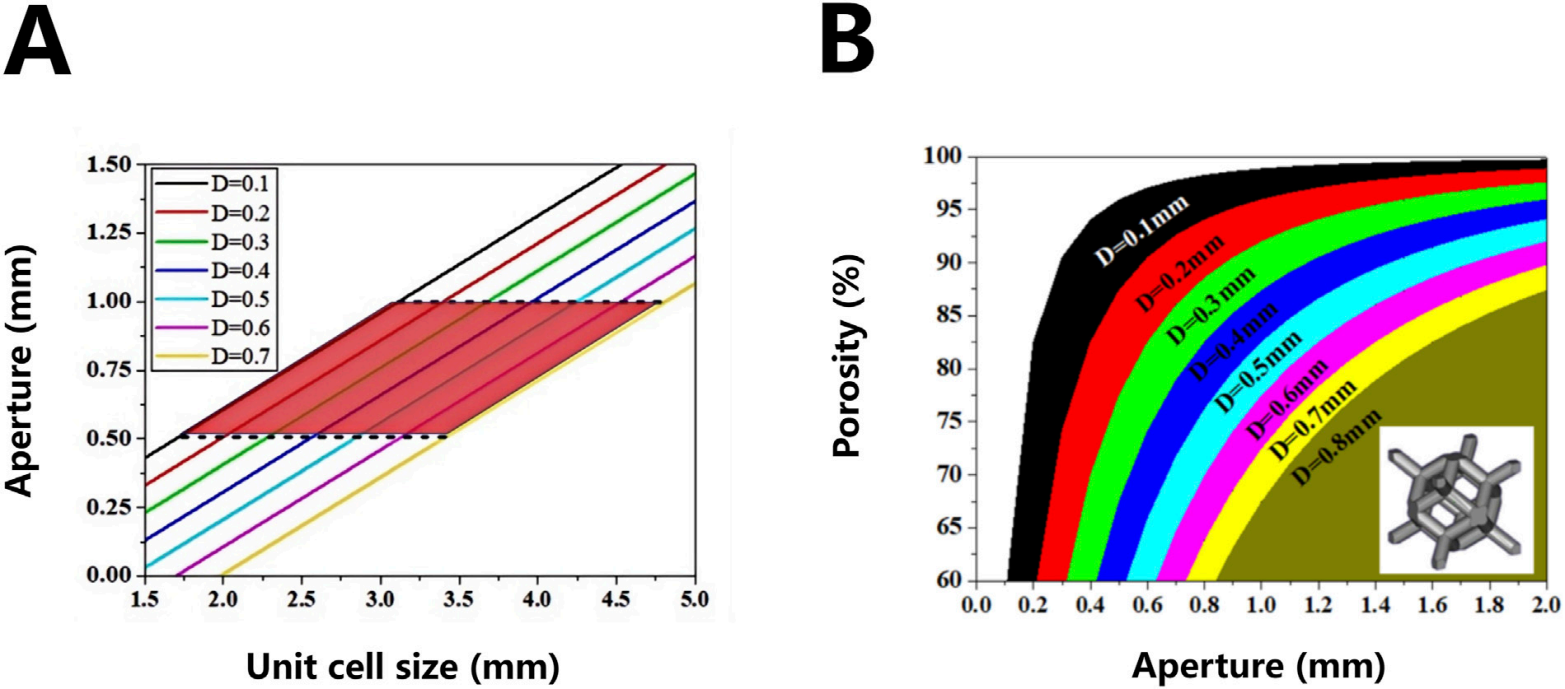
Correspondence between three-dimensional lattice units (unit cell size) and pore size of porous materials **(A)** and correspondence between pore size and porosity of rhombic dodecahedron porous structure **(B)**.

Where, H is the diameter of the inscribed circle of the three-dimensional pore structure unit (the aperture of the implanted material), M is the characteristic size of the three-dimensional pore structure unit (cell diameter, side length of the cube), and D is the diameter of the pore edge. Based on Equation 2, the corresponding relationship between the unit cell size M commonly used by model designers and the implant aperture can be obtained under different hole edge diameters, as shown in Figure 2A. Therefore, Equations 2, 3 will be used as another calculation basis for the structural design of porous tantalum implants.

On the basis of Formulas 1, 2, the formula for calculating the porosity of the porous body of the rhombic dodecahedron is calculated. The length of the hole edge commonly used by the model designer is replaced by a more clinically relevant hole diameter

$$\varepsilon =1-\left[1.38\times \frac{D^{2}}{{\left(M+D\right)}^{2}}-0.65\times \frac{D^{3}}{{\left(M+D\right)}^{3}}\right]$$ (4)

Based on Equation 4, the corresponding relationship between the pore diameter and the porosity of the implant can be obtained under different pore edge diameters, as shown in Figure 2B. This relationship will be used as an important calculation basis for the structural design of personalized porous Ta implants.

#### 2.1.2 Printing parameters of SEBM device

To ensure the repeatability of SEBM processing in this study, the parameters of SEBM equipment (Sailong Y150, Xi’an Sailong Additive Technology Co., Ltd., China) are as follows.

1. The temperature powder bed was preform with closed loop control (accuracy ±5 °C) to avoid thermal deformation;
2. Standardization of scanning parameters: current, speed, layer thickness and other parameters were fixed (scanning current 12 mA, speed 0.6 m/s);
3. Standardization of post-processing: high pressure sand blasting (0.5–0.6 MPa) with ultrasonic cleaning (2–4 h) to ensure that the pores were completely clean.

### 2.2 Materials preparation

The Ta metal powder was fabricated by Plasma Rotating Electrode Process (PREP) technology (Xi’an Sailong Additive Technology Co., Ltd., China). The microscopic morphology and chemical composition the Ta powder prepared by the PREP were shown in Figure 1A and Table 1. A rhombic dodecahedron (Figure 1B) was employed as the fundamental unit cell for printing tantalum block specimens with different porosity.

**TABLE 1:** Chemical composition of PREP tantalum powder and upper limit of standard impurity elements for medical tantalum materials (ppm).

Sample	C	O	N	H	Nb	Fe	Ti	Mo	Ni	Ta
ISO13782	100	150	100	15	1,000	100	100	200	100	Bal
PREP	10	60	10	5	-	46	4	70	1	Bal

^a^ PREP: plasma rotating electrode process.

### 2.3 Mechanical performance optimization of porous tantalum implants via selective electron beam melting (SEBM)

Porous Ta block specimens were fabricated using selective electron beam melting (SEBM) technology, with precise control over porosity and mechanical properties (Figure 1C).

**FIGURE 3:**
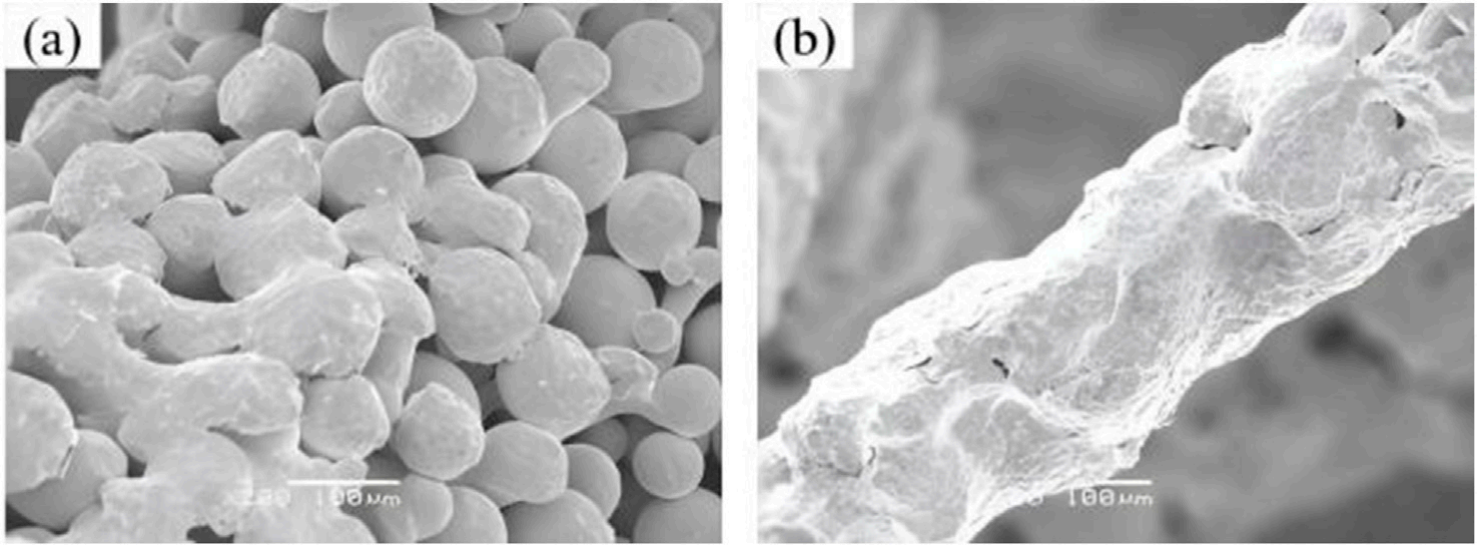
Microscopic morphology comparison of porous tantalum before and after powder removal. **(a)** Before cleaning; **(b)** After cleaning.

By high pressure injection, compressed air at 0.5–0.6 MPa pressure is utilized to entrain and eject tantalum metal powder, effectively purging unfused metal particles from both the porous tantalum matrix and internal pore channels. Multi-directional blasting from prescribed angles (visually verified through-pore directions) ensures comprehensive removal of residual powder within the porosity. Subsequent to high-pressure cleaning, any remaining trace particles in pore channels are further eliminated via ultrasonic agitation cleaning, with a duration typically ranging from 2 to 4 h (Figure 3). As there was no unified standard and method to identify whether the residual powder in 3D printing porous Ta was completely removed, weighing method was used to test the removal effect of powder. We suggested that if the weight difference of samples before and after cleaning is less than 0.1%, the powder residue could be considered removed from the internal pore network.

The dimensional and structural integrity of the porous networks were systematically characterized by scanning electron microscopy (SEM), which provided high-resolution observation into the pore morphology and surface topography.

To establish structure-property relationships, standardized mechanical test porous Ta specimens with tailored porosity (70%–85%) were fabricated and underwent comprehensive compression and bending tests to quantify the elastic modulus, yield strength, and failure mechanisms under simulated physiological loads.

### 2.4 Clinical case series

#### 2.4.1 Patient selection

This study prospectively enrolled three cases of complex spinal fusion surgery for cervical and thoracic vertebrae. All patients underwent preoperative planning using 3D printing technology and received personalized porous Ta implants.

Inclusion criteria: 1) Aged 18–70 years; 2) Determined to require vertebral fusion surgery for cervical and thoracic vertebrae upon evaluation through imaging methods such as X-ray, CT scan, or MRI; 3) In good overall health, capable of enduring the surgical and rehabilitation processes; 4) Without severe chronic conditions such as cardiovascular or cerebrovascular diseases, hepatic or renal insufficiency; 5) Fully informed about the treatment plan, associated risks, and expected outcomes, and provided written informed consent.

Exclusion criteria: 1) Allergic to tantalum (Ta); 2) Suffering from severe immune system disorders or systemic and localized infectious diseases; 3) Having mental illnesses, psychological problems, or other conditions that may affect treatment efficacy and patient compliance; 4) Unable to regularly participate in follow-up visits.

From March to May 2023, one male and two female patients who met the inclusion criteria were enrolled in our study. For the purpose of clearer presentation, each patient was assigned a unique identifier number.

This study protocol was registered and approved by the Ethics Committee of the First Affiliated Hospitalof Army Medical University, PLA (A)KY2023028. Each participant understood the procedures and precautions, and signed the informed consent form.

#### 2.4.2 Data acquisition and model reconstruction

**FIGURE 4:**
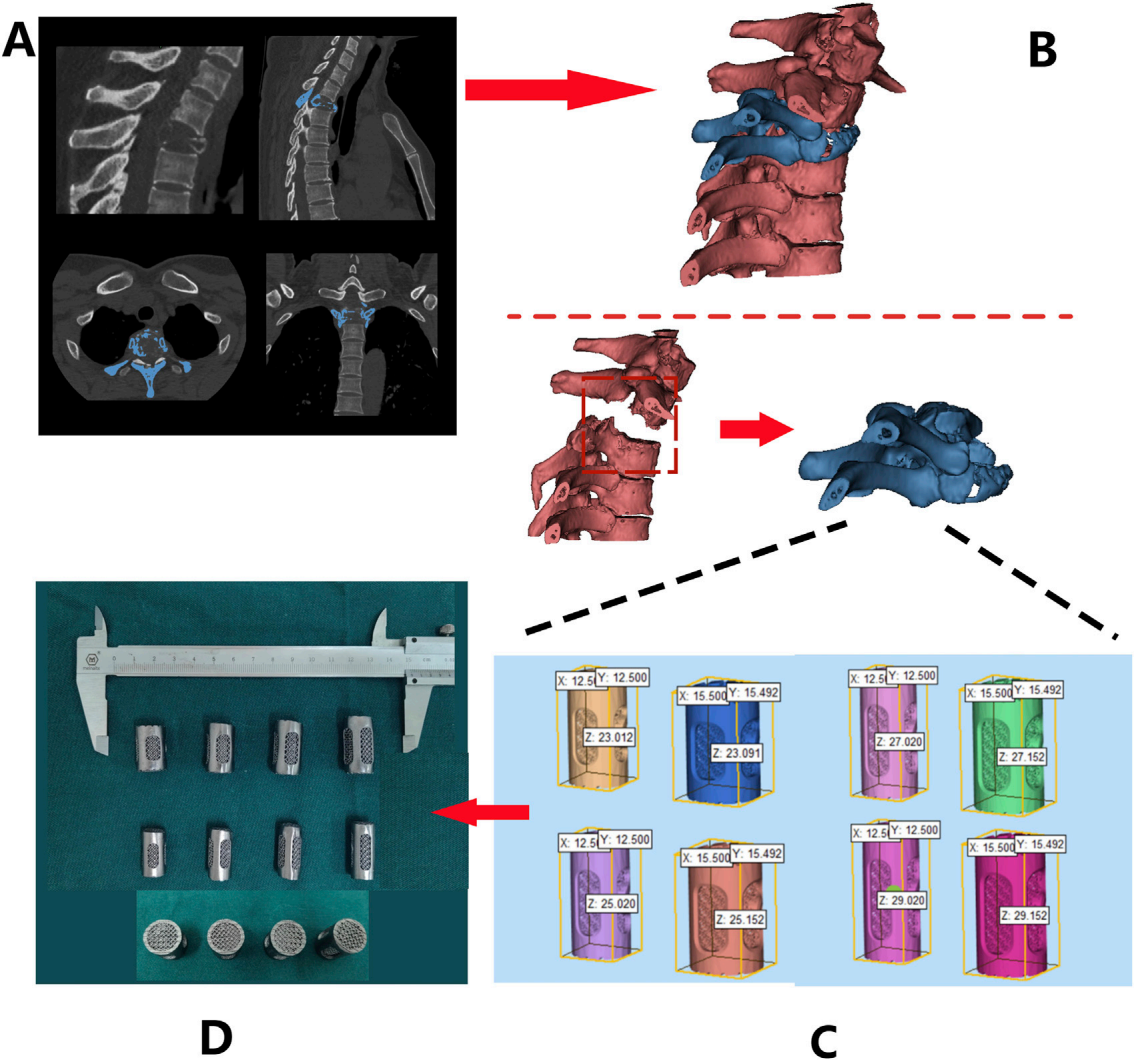
Design and fabrication process of 3D-printed porous tantalum vertebral body fusion implants (Patient 1 as an example): **(A)** data acquisition via thin-slice CT scanning; **(B)** three-dimensional reconstruction using MIMICS software based on CT data to generate a 3D model for analyzing lesion sites and other conditions; **(C)** implant design based on the 3D model; **(D)** final fabrication of implants.

The DICOM format of raw data from patients’ computed tomography (CT) scans of the spine (Figure 4A) were imported into MIMICS and 3-Matic software for segmentation and reconstruction (Figure 4B), generating 3D virtual models that provide intuitive anatomical references and assist in pre-surgical planning.

#### 2.4.3 Customized implant design

Design 3D-printed Ta implant based on the patient’s anatomical characteristics. These implants were characterized by multiple customizable properties, such as appropriately increased height and a 3° posterior convexity, restoring the normal spinal structure (Figure 4C). Screw holes and a roughened endplate contact interfaces that matches the patient’s endplate anatomy were designed as well (Figure 4D).

#### 2.4.4 Manufacturing and processing of porous tantalum implants

The Ta powder was additive manufactured according to the pre-operative design using SEBM (Sailong Y150 model, operating at 60 kV). After fabricating, redundant Ta powder removal was executed through high-pressure jet blasting, with parameters calibrated according to pore structure orientation and angle (Figure 4D).

#### 2.4.5 Surgical techniques

All patients underwent surgery under general anesthesia. Patients were placed in the prone position, with their heads fixed using a head frame. Surgical incisions were determined with the assistance of 3D electromagnetic navigation, followed by disinfection and draping of the surgical area. Soft tissues were carefully separated through the incision, while careful separation and protection of surrounding blood vessels and nerves were crucial to avoid unnecessary injury. The paraspinal muscles should be carefully detached from the posterior aspect to expose the posterior vertebral body and articular processes. Release of osteophytes were performed to attain a clean surgical field. After an appropriate implant was selected, the position and size should be confirmed under fluoroscopy. The entry points for the pedicle screws should be carefully exposed by lateral separation, and inserting screws bilaterally under 3D digital navigation and electrophysiological monitoring. Subsequently, Ti rods were implanted, with repeatedly tightening the locking screws. Autologous bone and allograft bone could be implanted into the intervertebral space as needed. Repeated C-arm fluoroscopy should be used to confirm the satisfactory positioning of the implants, internal fixation, and reduction of spine. The surgical area should be repeatedly irrigated with pulsatile lavage to prevent postoperative hematoma formation, followed by one or two indwelling rubber drainage tubes in the incision. Strict adherence to aseptic technique during the surgery is essential to reduce the risk of infection.

#### 2.4.6 Postoperative management

In the early postoperative period, the patient’s vital signs, including body temperature, blood pressure, heart rate, and respiratory rate, should be closely monitored. Regular changes of wound dressings should be performed to prevent infection and observe for any signs of bleeding, exudate, redness, or foul odor from the wound. Antibiotics should be administered postoperatively to prevent infection. If there is poor wound healing or a high risk of infection, antibiotic treatment should be extended, and the antibiotic regimen may be escalated as appropriate. Pain management should be tailored to the patient’s pain level, with routine use of ice packs, physical therapy, and topical or oral nonsteroidal anti-inflammatory drugs (NSAIDs). If pain symptoms persist, opioid medications may be added, but attention should be paid to potential side effects. Regular assessment of the patient’s limb sensation, motor function, and reflexes should be conducted to compare with preoperative status and evaluate the recovery of neurological function. If abnormalities such as increased limb numbness, weakness, or incontinence are observed, these may indicate nerve injury and require prompt intervention.

Maintaining proper positioning in the early postoperative period can promote wound healing and prevent spinal displacement. Patients are generally advised to lie flat on a firm mattress and perform axial rolling to avoid spinal torsion. When getting out of bed, patients should wear a brace to enhance spinal stability and protect the surgical site. Early mobilization, respiratory function exercises (such as balloon inflation and deep breathing), and assistance with sputum expectoration are encouraged. Early rehabilitation training helps to restore muscle strength and enhance spinal stability. Postoperative rehabilitation plans should be personalized based on the patient’s condition and may include muscle contraction and relaxation exercises, joint range-of-motion training, and balance training. As rehabilitation progresses, the intensity and difficulty of the exercises should be gradually increased. Postoperative dietary guidance should be provided to ensure a balanced nutritional intake that promotes wound healing and overall recovery.

During the follow-up period, regular clinical evaluations and imaging examinations are required. These evaluations encompass assessing the patient’s pain condition using the Visual Analog Scale (VAS) score, as well as examining whether there is any limitation of movement or postoperative complications. Imaging examinations, primarily conducted through X-rays or CT scans, are mainly aimed at observing the position and stability of the prosthesis, along with its osseointegration (bone fusion) with the adjacent endplates.

## 3 Result

### 3.1 Pore structure and surface property of porous tantalum

**FIGURE 5:**
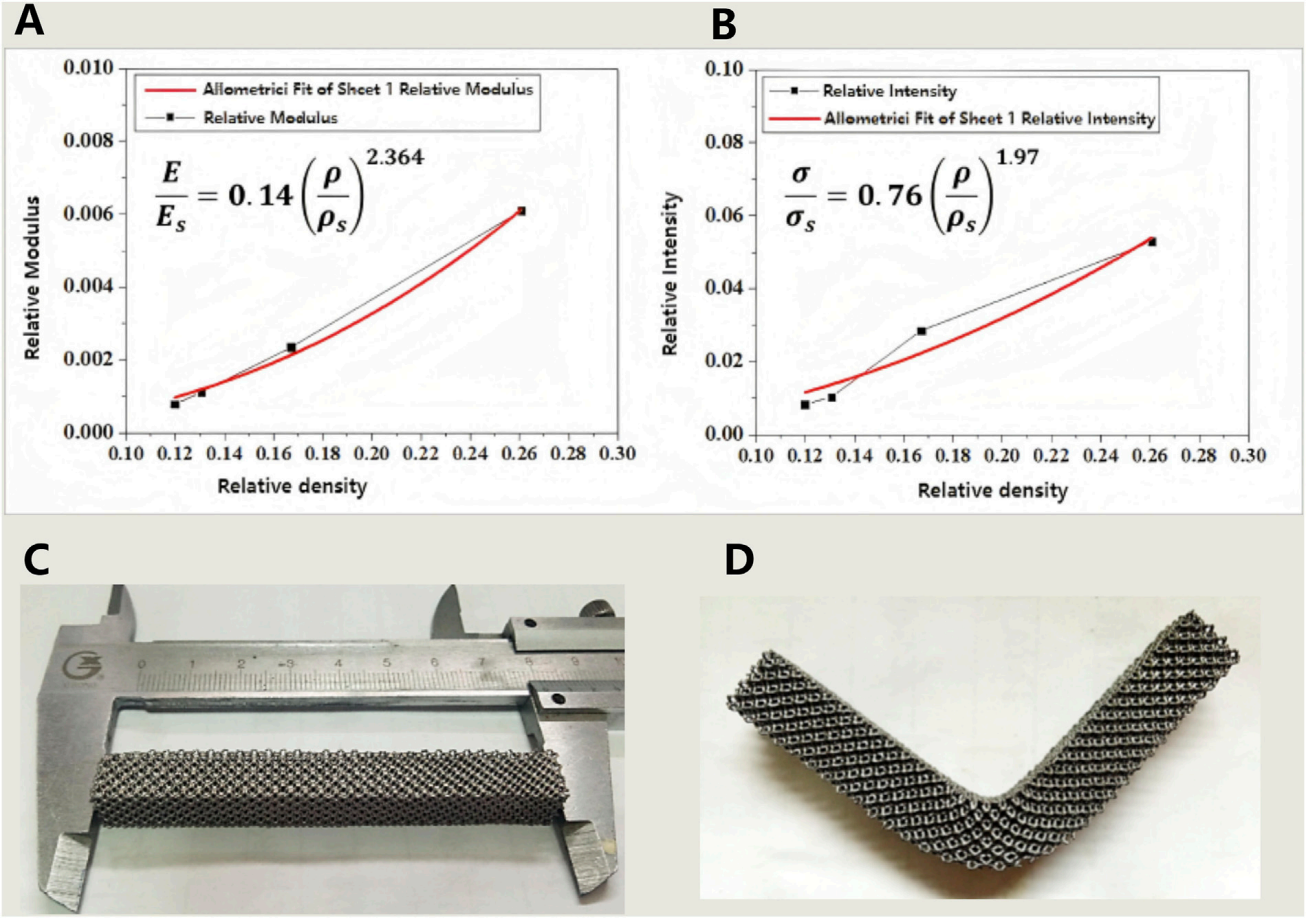
**(A,B)** The relationship between relative density and modulus **(A)** as well as strength **(B)** of porous tantalum fabricated by SEBM 3D printing; **(C,D)** Results of the three-point bending strength test for porous tantalum specimens (specimen dimensions: 10 × 10 × 80 mm): **(C)** Physical specimen before testing; **(D)** Physical specimen after testing.

SEM was employed to observe the microstructure morphology of various porous tantalum samples (Figure 5A). The pore structures of the porous Ta samples were intact, with smooth pore strut surfaces and no signs of sticking or adhesion of unmelted powder. Observation of the microstructure morphology of the internal channels of the samples revealed that the internal quality was consistent with the external quality, fully validating the reliability of the porous Ta implant fabrication process (Figure 5B).

### 3.2 Evaluation of mechanical properties

**FIGURE 6:**
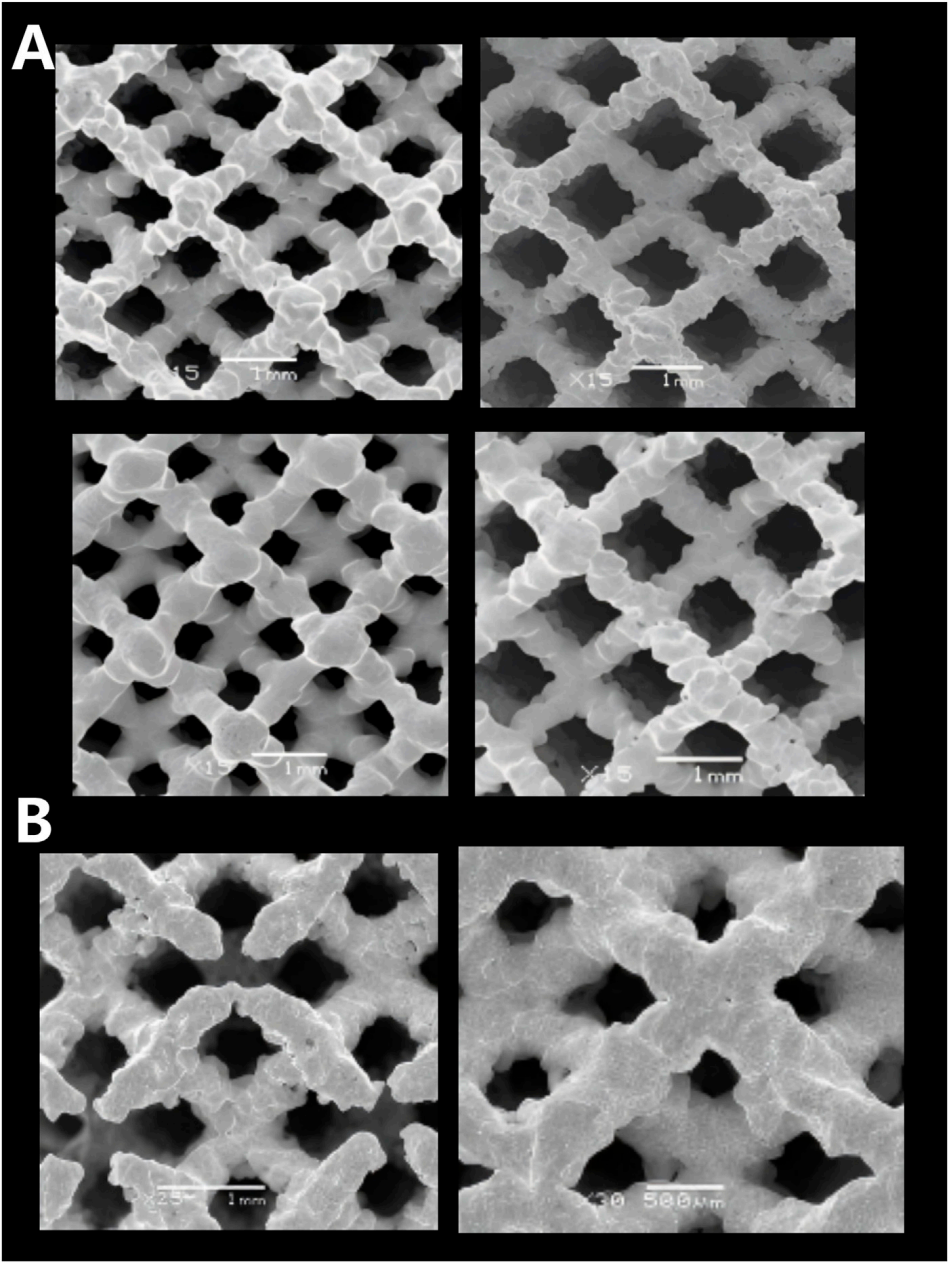
Microscopic morphologies of porous tantalum samples with different specifications fabricated by SEBM. **(A)** Surface pores; **(B)** Internal pores.

The compressive properties of porous tantalum were measured, with the results shown in Table 2. For porous metallic materials, the Gibson–Ashby models could accurately predict the correlation between relative density and strength/modulus. The mechanical properties of porous Ta were fitted, demonstrating that the mechanical properties of SEBM 3D-printed porous tantalum conformed to the models, while with differences in the power exponent term compared to the classical models, which may arise from the unique surface state. Nevertheless, the two equations in Figures 6A,B will still serve as crucial references and data support for the structural design of porous Ta implants.

**TABLE 2:** Mechanical properties of porous tantalum with different porosity.

Sample	Porosity (%)	Elastic modulus (GPa)	Yield strength (MPa)	Plateau stress (MPa)
A	45.54	4.71	33.46	103.88
B	73.92	1.28	13.75	23.89
C	83.29	0.49	7.42	10.19
D	86.94	0.23	2.70	4.62
E	88.02	0.17	2.16	3.99

Bending resistance of porous Ta samples was tested (Figures 6C,D). The test results indicate that the porous Ta prepared in this study exhibits excellent bending resistance (strength of 48 MPa), without any structural damage occurring after deformation.

### 3.3 Case series

All three patients (including two females and one male) successfully underwent surgeries and all recovered satisfactorily after surgery (Table 3).

**TABLE 3:** General statistics and surgical data of all patients.

Patient	Age	Gender	Position	Cause	Diagnosis	Follow-up (m)	Surgical duration (min)	Blood loss (mL)	Los (days)	VAS score	
										Pre-	Post-
1	59	female	atlanto-axial joint	No obvious cause	Old atlantoaxial dislocation	25	188	300	33	6	2
2^a^	46	male	T6	multiple myeloma	pathological fracture	24	450	785	36	7	1
3^a^	49	female	T2	GCT of bone	pathological fracture	24	525	1,000	21	7	2

^a^ Indicated that the patient had received a blood transfusion.

**FIGURE 7:**
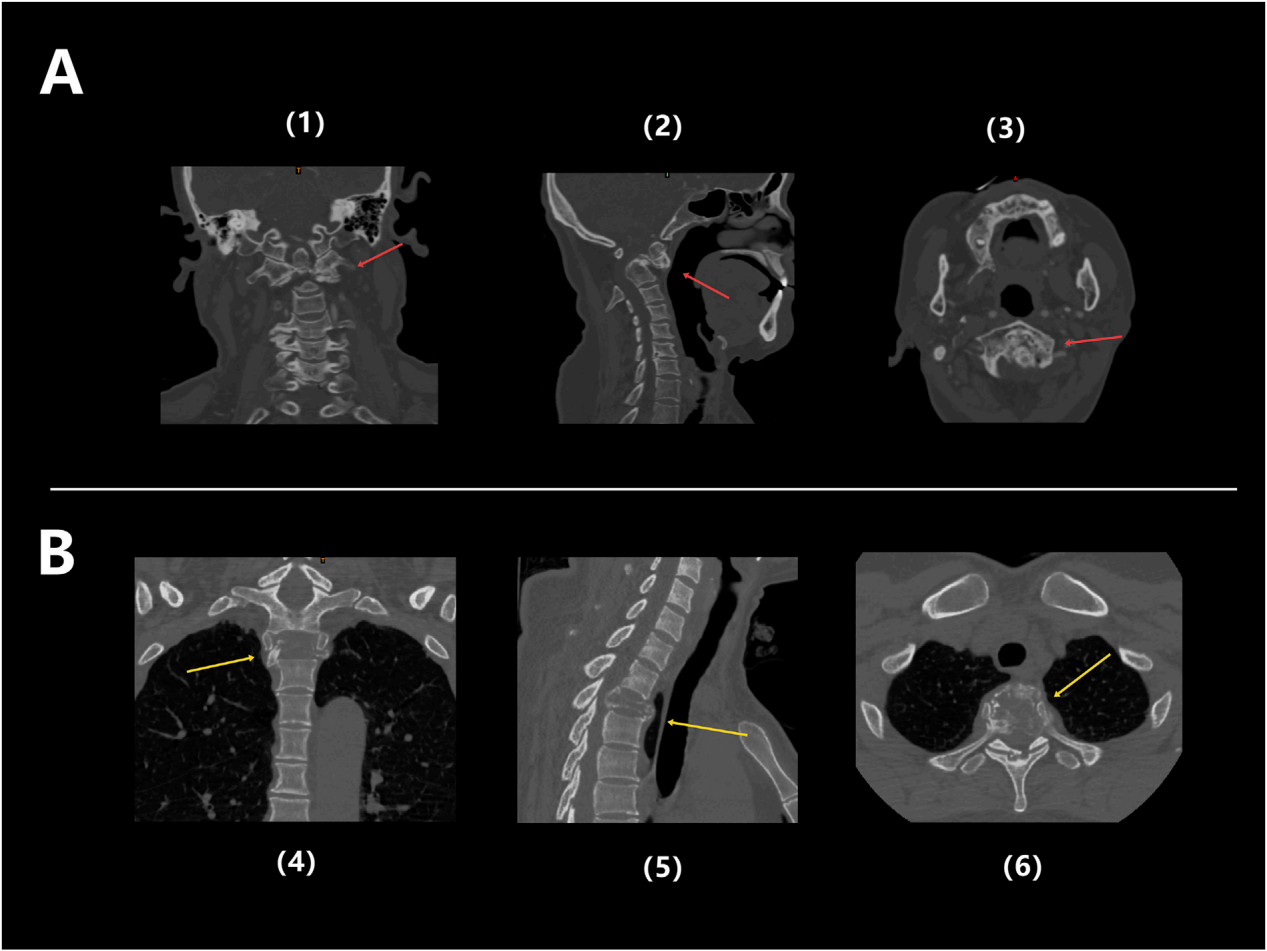
**(A)** Preoperative CT images of Patient 1: Coronal (1), Sagittal (2), and Axial (3), showing obvious structural abnormalities and irregular shapes in the atlantoaxial joint, with the backward tilted axis and significantly compressed and narrowed local spinal canal, and spinal cord compressed (red arrow); **(B)** Preoperative CT images of Patient 3: coronal (4), sagittal (5), and axial (6), showing significant bone destruction in the T2 thoracic vertebrae, accompanied by pathological fractures (yellow arrow).

**FIGURE 8:**
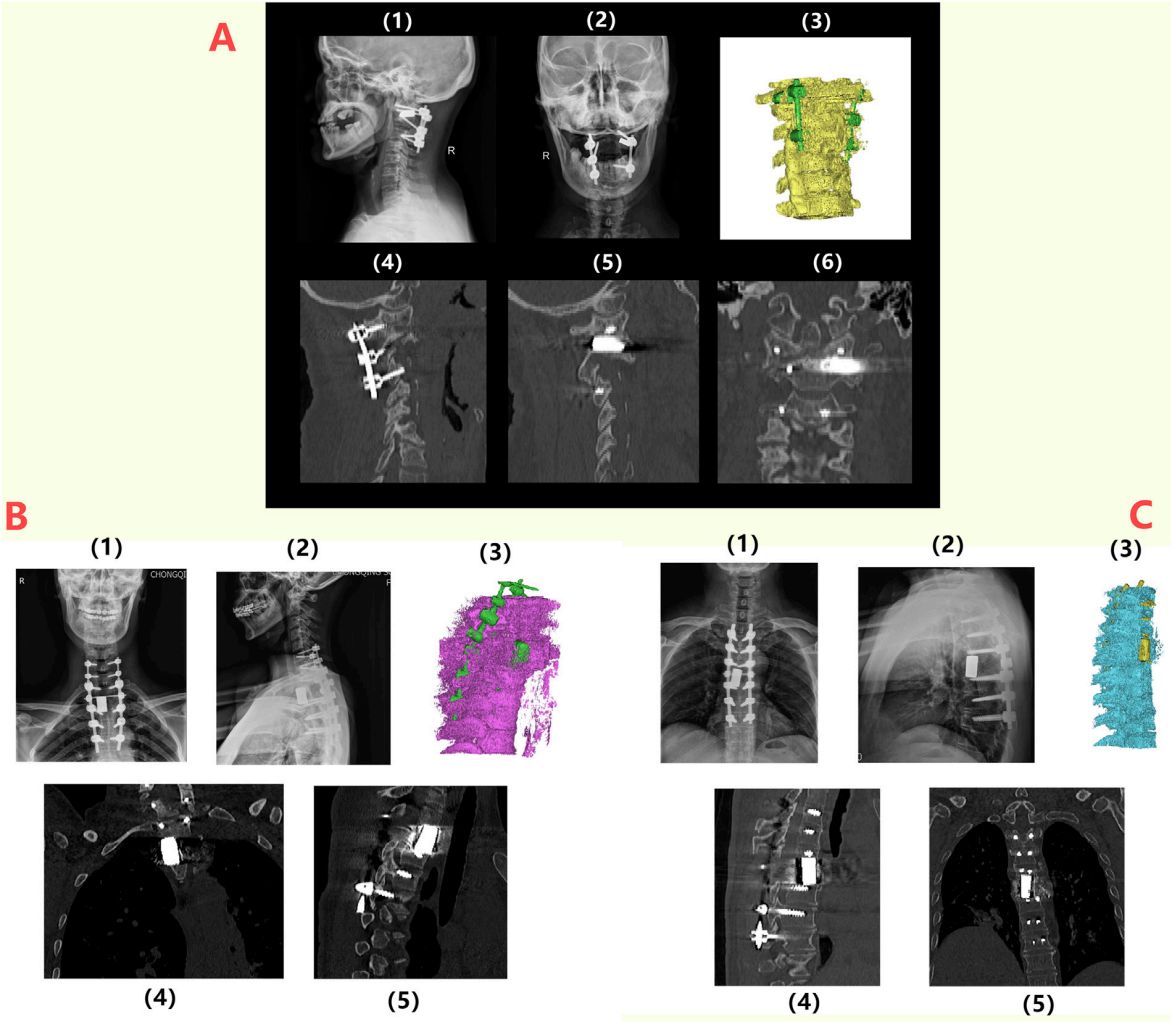
Postoperative radiographic follow-up of patients: **(A)** 1/2: Lateral and anteroposterior X-ray images of the spine of Patient 1 at 6 months postoperatively, indicating the proper positioning of the prosthesis and internal fixation; 3/4/5/6: CT images and three-dimensional reconstruction at 6 months postoperatively, showing no loosening or displacement of the prosthesis and internal fixation; **(B)** 1/2: Lateral and anteroposterior X-ray images of the spine for Patient 3 at 6 months postoperatively, indicating the proper positioning of the prosthesis and internal fixation; 3/4/5: CT images and three-dimensional reconstructions at 6 months postoperatively, showing no loosening or displacement of the prosthesis and internal fixation; **(C)** 1/2: Lateral and anteroposterior X-ray images of the spine for Patient 2 at 6 months postoperatively, indicating the proper positioning of the prosthesis and internal fixation; 3/4/5: CT images and three-dimensional reconstructions at 6 months postoperatively, showing no loosening or displacement of the prosthesis and internal fixation.

Patient 1 is a 59-year-old female diagnosed with old atlantoaxial dislocation. Cervical CT showed irregular morphology of the atlantoaxial joint, posterior tilt of the axis and obvious compression and narrowing of the spinal canal with compression of the spinal cord (Figure 7A), leading to unstable walking in both lower limbs. After received personalized 3D printing of tantalum metal prosthesis C1/2 joint space implantation and allogeneic bone graft fusion with pedicle screw internal fixation, the patient’s symptoms were significantly improved. Postoperative imaging during follow-up showed satisfactory fixation and prosthesis position, without significant looseness or displacement observed (Figure 8A).

Patient 2 was a 46-year-old male diagnosed with isolated plasmacytoma (T6-SPB) of T6 vertebra and adnexa accompanied by pathological fracture of T6 vertebra, complaining bilateral rib pain for more than 7 months. After PCD chemotherapy, the patient underwent *En Bloc*/TES resection and 3D printed tantalum metal prosthesis reconstruction. Regarding the detailed clinical information of Patient 2, please refer to 3.3.1 Typical Cases.

Patient 3 was a 49-year-old female diagnosed with T2 vertebral giant cell tumor GCT accompanied by pathological fractures (Figure 7B). After five cycles of neoadjuvant chemotherapy with denosumab, the patient underwent extensive resection of T2 vertebral giant cell tumor via posterior approach, followed by 3D printed tantalum metal prosthesis reconstruction with internal pedicle screws fixation of C6-T5 (Figure 8C). The surgery went smoothly, with satisfactory postoperative recovery and significant improvement in pain symptoms. Postoperative imaging during follow-up showed satisfactory fixation and prosthesis position, with no significant looseness or displacement observed.

The duration of the operations ranged from 188 to 525 min, with an average of 387.7 min. Intraoperative blood loss ranged from 300 to 1,000 mL, with an average of 695 mL. The length of hospital stay was between 21 and 36 days, averaging 30.0 days (see Table 3). Two patients (Patient 2 and Patient 3) received blood transfusions postoperatively. All patients’ body temperatures returned to normal within 1 day after surgery. All incisions healed well, meeting Grade A healing criteria.

The follow-up period ranged from 24 to 25 months, with an average of 24.3 months. As of the most recent follow-up, all patients showed significant improvement in pain symptoms (VAS score). Notably, no serious complications such as postoperative infection, prosthesis loosening, or vascular nerve injury occurred in any patient.

#### 3.3.1 Typical case - patients with isolated plasmacytoma of T6 vertebrae and adnexa

**FIGURE 9:**
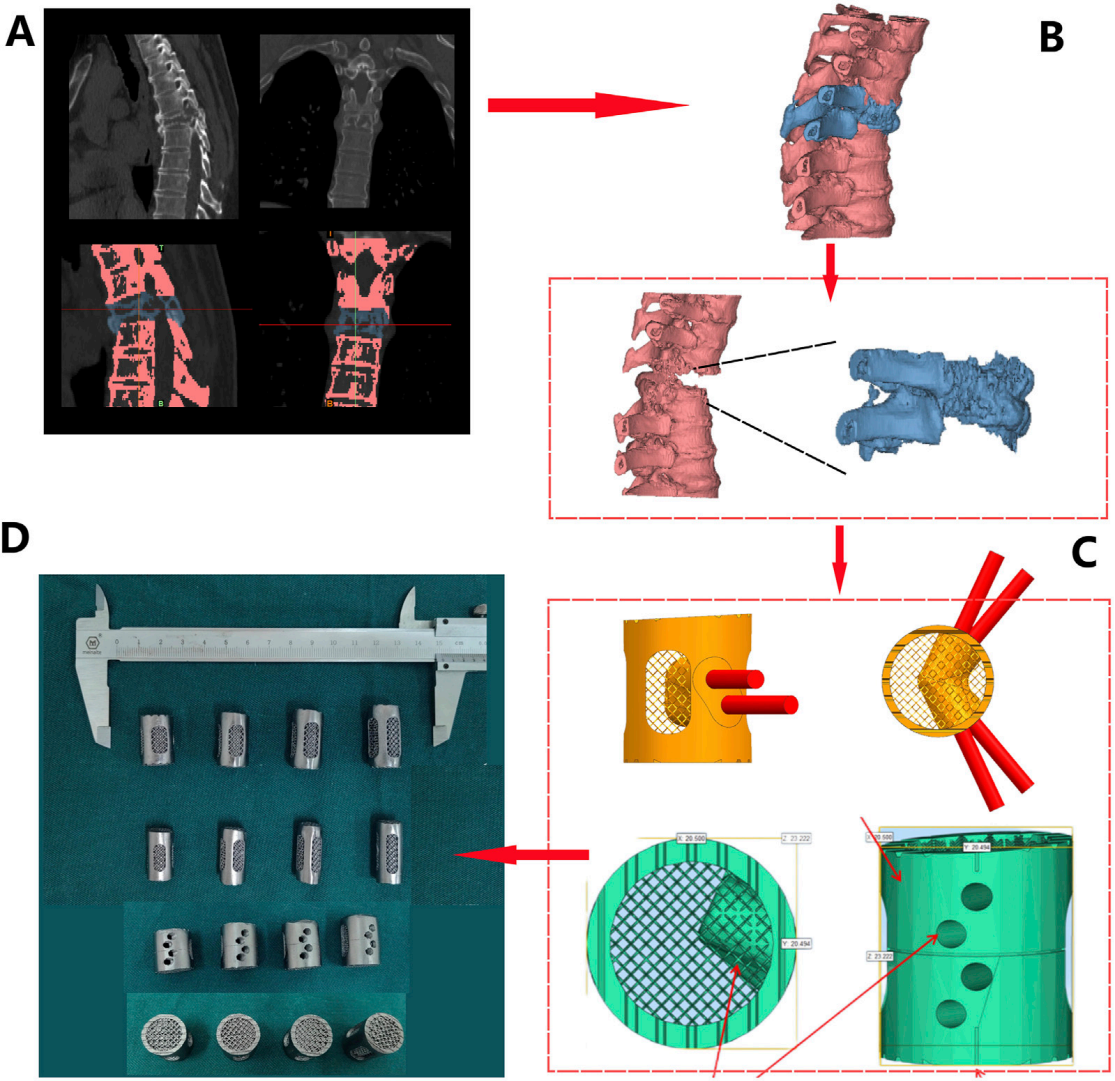
Prosthesis design and fabrication process for patient 2: **(A)** CT Scan Images: obvious bone destruction in the T6 vertebral body, with compression and deformation of the vertebral body (1/2). T6 and adjacent vertebrae were differentiated by threshold adjustment (3/4); **(B)** Three-dimensional reconstruction based on CT data (red - normal vertebrae, blue - T6); **(C)** Prosthesis design was conducted based on measurement, including a 3° retroversion angle (1), 30° and 45° screw holes (2), polished lateral surface design (3), and roughened superior and inferior surfaces (4); **(D)** Four sizes of porous tantalum prostheses with varying heights (21/23/25/27 mm) were fabricated via 3D printing.

A 46-year-old male patient (Patient 2) presented with a chief complaint of intermittent and gradually worsened pain in bilateral rib for over 7 months. Physical examination indicated no obvious deformity or significant limitation of movement of his spine. Below the xiphoid process plane, hypoesthesia was observed in the trunk, abdomen, perineum, and superficial areas of the lower extremities, with marked symmetrical numbness in both feet, calves, and the mid-thigh regions. CT scan revealed bone destruction of the 6th thoracic (T6) vertebra accompanied by a compressive fracture, with an increasing density of surrounding soft tissue, suggesting a possibility of neoplastic metastasis (Figure 9A). Preoperative pathological biopsy and immunohistochemistry supported the diagnosis of plasmacytoma.

Preoperative spinal stability assessment confirmed spinal instability, with a Spinal Instability Neoplastic Score (SINS) of 15, providing clear indications for surgery. Due to a favorable clinical prognosis with a relatively long survival time of plasmacytoma, after a multidisciplinary discussion involving spine and bone tumor surgeons, surgical procedure was decided to preserve spinal stability and spinal cord function. The surgical plan involved resection of the T6 vertebral body followed by reconstruction using a 3D-printed porous Ta implant. Due to the anatomical complexity associated with *en bloc* resection of the entire vertebral segment to achieve a clean surgical margins, personalized surgical planning and implant design were crucial to reduce total time of surgery and intraoperative blood loss.

Following a 3D reconstruction of the T6 vertebral body and its surrounding vertebrae using Mimics 21.0 software, prosthesis design was performed (Figure 9B). Using the T5 and T7 vertebrae as references, we simulated and designed a T6 porous tantalum implant, featured a 3° kyphotic angle to enhance spinal stability. Four prostheses with gradient heights (21 mm/23 mm/25 mm/27 mm) were designed (Figure 9C), with screw holes on both sides of the prosthesis. The upper and lower ends were roughened to create a serrated surface for better contact. According to the preoperative design and in combination with weight reduction achieved through the porous structure, the prosthesis was fabricated using Ta powder (Figure 9D).

**FIGURE 10:**
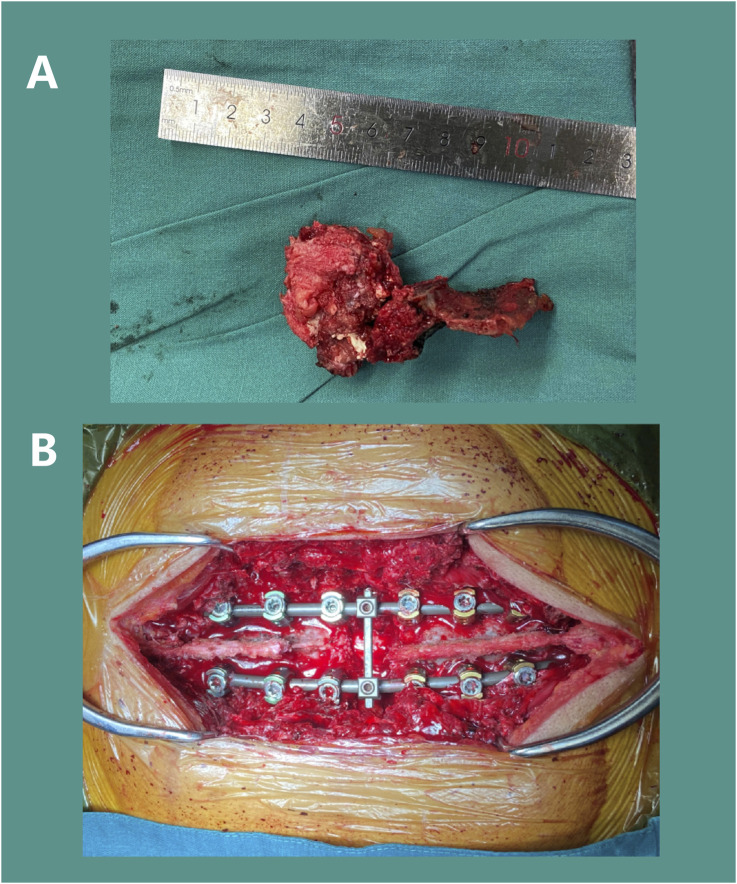
Surgical Procedure: Resection of the T6 tumor mass **(A)** and installation of pedicle screw-rod internal fixation **(B)**.

After satisfactory general anesthesia, the patient was placed in the prone position and properly secured. Under 3D electromagnetic navigation, pedicle locations of the T3, T6, and T9 was marked at the skin. Routine disinfection and draping were performed. An approximately 25 cm posterior midline incision was made from T2 to T10. After incising skin and subcutaneous fascia, the paraspinal muscles were dissected along the spinous processes to fully expose the posterior spinal column structures, including the spinous processes, laminae, articular processes, and pedicles of the T3-9, with thorough hemostasis. Twelve appropriately sized pedicle screws were inserted along the bilateral T3-9 (excluding T6) pedicles and confirmed to be in satisfactory positions by using C-arm. Then the left T6 costovertebral joint was exposed, and the rib was dissected subperiosteally. The parietal pleura should be carefully protected. The head of rib was resected approximately 5 cm by ultrasonic osteotome, and the costovertebral joint and rib were removed with careful hemostasis. The intercostal nerves and vessels were exposed, ligated, and divided. The same procedure was repeated on the right side. The T5 spinous process and bilateral T6 posterior column bone were osteotomized. The remaining bone was removed with a rongeur, revealing full exposure and pulsation of the T6 spinal dural sac. The tumor tissue was completely resected (Figure 10A). The T6 dural sac was fully freed within the spinal canal, showing a dural injury and cerebrospinal fluid (CSF) leak at the initial portion of the left T6 nerve root, which was microscopically sutured. Hemostatic gauze was used for hemostasis, and the spinal cord was covered and protected. Gauze was packed into the bilateral T6 paravertebral posterolateral spaces to protect the pleura. Under 3D navigation, a puncture needle was inserted along the T6 pedicle to confirmed a appropriate position. 2 mL bone cement was mixed and injected into the T6 vertebra to reinforce vertebral body and control bleeding. After complete exposure of the paravertebral space, the T6 pedicle was removed. Extensive gauze packing assisted in separating and protecting the parietal pleura. The lateral dissection should be extended to the anterior vertebra, while the separation from the aorta should be confirm by fingers encircling the lateral edge of the vertebral body and ensuring the integrity of the anterior thoracic aorta, superior vena cava, and esophagus. After confirming bilateral convergence, a pre-bent and appropriately sized Ti rod was inserted to fix the left pedicle screws and lock the T5-7 gap. The intervertebral discs adjacent to T6 were resected. The T6 dural sac was fully freed within the spinal canal. Completely remove the whole tumor and the surrounding anterior and posterior longitudinal ligaments. The surgical site was coagulated for hemostasis and thoroughly irrigated. An appropriately sized prosthesis (15 mm in diameter, 25 mm in height) was selected and inserted into the anterior and middle column positions. A pre-bent, appropriately sized Ti rod was inserted to fix the right pedicle screws, while the prosthesis was sequentially compressed and locked. Repeated C-arm fluoroscopy was used to confirm the position of prosthesis and reliable transverse connection fixation (Figure 10B). Then the surgical area was irrigated, followed by completely hemostasis. The resected autologous rib bone was sufficiently implanted around the prosthesis. A 360-degree exploration around the T6 spinal cord indicated a normal dural sac pulsation and no significant leakage of CSF. C-arm fluoroscopy showed satisfactory bone graft reconstruction and internal fixation. Two negative pressure drainage tubes were placed beside each bilateral incision and fixed. The incisions were closed layer by layer, and sterile dressings were applied. The patient’s vital signs were stable postoperatively and safely back to the ward.

The patient recovered rapidly and strictly wore a cervicothoracic brace for protection for 3 months before sitting up and standing with assistance. To prevent infection, antibiotic treatment was prescribed, while oral and topical analgesics effectively alleviated postoperative pain symptoms. The wound healed very smoothly, and the sutures were successfully removed 14 days after surgery. Spinal X-rays and CT scans conducted 2 years postoperatively showed satisfactory positioning of the T6 implant and internal fixation, with no signs of displacement or loosening (Figure 8C). During the latest follow-up, the patient reported good recovery of spinal function without any significant discomfort.

## 4 Discussion

Combining the verification of the microstructure, mechanical properties, and cytotoxicity of SEBM 3D printed customized porous tantalum implants, as well as the clinical application and mid-term efficacy of porous Ta implants in complex cervical and thoracic vertebral fusion, this study comprehensively elaborates on the rational application of additive manufacturing and porous Ta. Correspondingly, this study has indeed confirmed the safety and superiority biomechanical properties of porous Ta. Meanwhile, the clinical case series confirms the superiority and safety of personalized porous Ta implants in complex cervical and thoracic vertebral fusion surgeries.

Currently, materials such as Ti and PEEK have great potential to replace autogenous bone graft as the choice of intervertebral fusion implants (Zhang et al., 2023a). However, as biologically inert materials, both Ti and PEEK lack excellent osteogenic properties and bone integration with the host bone, greatly increasing the probability of long-term implant failure (Wang et al., 2023). A retrospective cohort study of Segi et al. compared the outcomes of 101 patients who underwent single segment posterior lumbar interbody fusion (PLIF) using three-dimensional porous titanium alloy (3DTi) cavities and polyetheretherketone (PEEK) cages (Segi et al., 2025). Their results showed that within 1 year after surgery, the rate of vertebral endplate cyst (VEC) and cage subsidence after implantation of both materials reached over 40%. In contrast, porous tantalum metal cages have significantly lower rates of VEC occurrence with better bone integration in the early postoperative period (3 months) compared to titanium coated PEEK cages, while achieving better intervertebral stability (Segi et al., 2023). As a bioactive material, porous Ta has been proven to have better properties in promoting bone regeneration. However, although it has been widely used, its clinical application in spinal surgery still requires further research. In spinal fusion, a good bone integration between the implant and the vertebrae is crucial for a successful surgical prognosis. Zhang et al. demonstrated excellent bone integration and biocompatibility of porous tantalum fusion cages supported by 3D printing and CVD through animal models (Zhang et al., 2023b), and affirmed the unique advantages of 3D printing in achieving complex structural design, low-cost manufacturing, and personalized customization; The research results of Liang et al. showed (Liang et al., 2023) that the elastic modulus of porous Ta fusion device is similar to that of cancellous bone, which can effectively reduce stress shielding. Meanwhile, porous Ta has superior biocompatibility and osteogenic potential, promoting better bone growth. The osteogenic properties of tantalum have also been widely validated. The *in vivo* experiment in rabbit model showed that porous tantalum is non-toxic, while has good biocompatibility and osteoinductive properties (Wang et al., 2015). Zhang et al. confirmed in vivo experiments (Zhang et al., 2021) that tantalum metal could enhance early osteogenesis both *in vitro* and *in vivo* by constructing a Nanotube decorated hierarchical tantalum scaffold. Many systematic reviews has also demonstrated that Ta has excellent osteoinductive properties and is a promising material for bone tissue engineering (Yu et al., 2024).

In our study, porous tantalum fabricated by SEBM had superior biomechanical properties. Especially compared with traditional orthopedic implant materials, the elastic modulus of porous tantalum (0.17–4.71 GPa) is highly similar to that of cancellous bone (3–4 GPa), while titanium alloy (110 GPa) and PEEK (3.8 GPa) have significant differences from cancellous bone (Gao et al., 2023). The characteristic of porous tantalum can effectively reduce stress shielding. At the same time, porous tantalum has a bending strength of up to 48 MPa, significantly higher than PEEK (20–30 MPa), making it have good fatigue resistance and more suitable for withstanding dynamic loads such as cervical flexion, extension, and rotation (Toro et al., 2022). The good platform stress (10–23 MPa) of porous tantalum with a porosity of 70%–85% provides suitable mechanical stimulation for bone cells. PEEK, due to its non porous structure, has a platform stress of up to 50 MPa, which inhibits bone tissue growth (Romanos et al., 2016). The results of *in vitro* cytotoxicity test also confirmed its safety. MTT analysis showed that the cell survival rate of all experimental groups was more than 75%, indicating a good biocompatibility and low cytotoxicity, providing a safe environment for bone in-growth and integration. Three patients were followed up for an average of 24.3 months without any serious events, while their radiological images showed that the porous Ta implants were well bonded to the bone interface without loosening.

Both cervical deformities and thoracic vertebra tumors are rare and complex, and their surgical treatment could be very challenging even for experienced orthopedic surgeons (Kaneyama et al., 2014). Although there were some literature about the clinical application of 3D printing and navigation technology in cervical and thoracic spine surgery (Sugawara et al., 2018) (Saß et al., 2019), there was still no literature reporting the application of 3D printed Ta metal implantation in complex cervical and thoracic deformities and tumor reconstruction, which cannot provide sufficient reference for clinical applications. Compared with the relatively simple anatomical features of the lumbar spine, the cervical spine is surrounded by numerous vital nerves and vascular tissues (Chen et al., 2023) (Echt et al., 2020). Meanwhile, the cervical spinal cord controls many key functions of the human body, which may lead to serious complications such as limb paralysis and sensory disorders if any slight compression or injury occurred (Chen et al., 2007). The thoracic vertebrae are connected to the ribs, limiting the space of surgery. Moreover, the blood supply to the thoracic spinal cord is relatively poor, which may lead to ischemic injury that can seriously affect the recovery of neurological function if the blood supply is damaged during surgery (Rustagi et al., 2020). In addition, the relatively thinner pedicle of thoracic vertebrae requires higher surgical skills when performing pedicle screw fixation since a slight deviation may damage the spinal cord or surrounding blood vessels and nerves (Jeger et al., 2023).

Customize surgical planning and 3DP navigation according to the different anatomical characteristic of each patient could be highly valuable in complex cervical and thoracic spine surgeries. In their retrospective study, Wu et al. used personalized 3D printing patient-specific navigation templates to treat unstable Atlas Fractures (Wu et al., 2021), with the assist of a screw assisted system, successfully restored the occiputocervical induction (OCI) to normal, reducing the incidence of complications while completing occiputocervical fusion. Sugawara et al.'s multicenter clinical study showed that using 3D/multi platform imaging technology for intraoperative pedicle screw (PS) cervical and thoracic navigation can effectively improve the accuracy of PS insertion and reduce the time of spinal fixation surgery and surgeon radiation exposure (Sugawara et al., 2018). In Hunn et al.'s case series (Hunn et al., 2020), the use of customized 3D printed prostheses to reconstruct the C2 vertebral body was described, where the advantages of 3D printed customized prostheses in complex spinal surgery were detailed. In our study, we achieved a great therapeutic effect by conducting a personalized design, effectively improving surgical efficiency and safety. All three patients with old atlantoaxial dislocation (Patient 1) and complex thoracic tumor surgery (Patients 2 and 3) underwent smooth surgical procedures. For patient with atlantoaxial dislocation (patient 1), the anatomical structure was recognized in advance through personalized preoperative planning, which effectively avoided the intraoperative injury of important nerves and blood vessels, improving the operation efficiency (time of surgery: 188 min) and eventually preventing postoperative blood transfusion (intraoperative blood loss: 300 mL). Giant cell tumor of bone (GCTs) is an aggressive benign primary bone tumor, where spine accounts for about 2.7%–6.5% of all bone GCT (Niu et al., 2012). For thoracic GCT, early and complete *en bloc* resection can achieve a lower recurrence rate (Mayfield et al., 2022), while the complexity of surgery and long operation time can bring great challenges to surgeons. In our case (patients 2 and 3), precise preoperative planning can achieve a clean surgical boundary while minimizing the operation time and intraoperative blood loss. Meanwhile, porous Ta prosthesis with good osseointegration also ensured that patients did not have any prosthesis-related complications within 2 years after operation.

Porous tantalum has significantly better properties than traditional Ti alloys or PEEK in terms of high porosity (75%–85%), low elastic modulus (close to trabecular bone), and high surface friction coefficient (0.80–0.74) (Yuan et al., 2022). Its three-dimensional interconnected pore structure could effectively promote bone in-growth and osteogenesis (Liu et al., 2015), providing an ideal microenvironment for cell proliferation and tissue regeneration. In addition, the chemical inertness of Ta enables it to exhibit extremely high corrosion resistance in internal environment, resulting in better biocompatibility (Fernández-Fairen et al., 2019). In our study, all three patients showed significant improvement in symptoms during follow-up without prosthesis loosening or infection. Advanced 3D printing technology enabled us to design and fabricated porous Ta prosthesis according to the needs of different patients. During the process of designing, the overall anatomical characteristics were carefully taken into consideration. For two patients with thoracic tumors (patients 2 and 3), we designed the prosthesis with 3 degrees of kyphosis to fit the shape of the spine. Meanwhile, the upper and lower surfaces of the prosthesis were roughened to achieve better attached to the vertebral body and promote the bone in-growth of interface. The specially designed screw fixation hole with placement angles of 30° and 45° can adapt to different intraoperative conditions and greatly improve the operation efficiency and safety.

It is important to note, however, that the present study has certain limitations. Firstly, this study did not fully demonstrate the osteogenic ability of porous Ta through *in vitro* and *in vivo* animal model experiments, which seems to make the results less convincing. However, the reason is that the biological safety and osteogenic superiority of porous Ta have been extensively studied and demonstrated, while our previous researches on the basic and clinical applications of porous Ta is already very sufficient (Supplementary Material 1). Meanwhile, the main purpose of this study is the clinical application details of personalized porous Ta prostheses in complex cervical/thoracic spine surgeries and provide reference for future research and surgical technique improvement. Secondly, the sample size of the case series included only 3 cases, which may limit the generalizability. The reasons might include that there were few clinical cases of complex cervical thoracic fusion surgery (such as tumor resection and severe deformity). Meanwhile, this study was an exploratory experiment to verify the feasibility. Although preliminary results indicated good mid-term efficacy in complex cervical/thoracic fusion, further validation of its effectiveness and safety through larger cohort studies is needed. Secondly, the follow-up period was relatively short, still requiring longer observation to evaluate the long-term outcomes and potential complications of the prosthesis. However, as this study focused on introducing the details of the entire process from 3D printing manufacturing to application and its mid-term clinical efficacy, we will also continue to follow up with all patients to further evaluate long-term prognosis. Finally, in the future, while expanding the sample size, it is necessary to add suitable control groups for further comparison with traditional surgical methods, in order to enhance the persuasiveness of the research results.

In conclusion, this study demonstrated the feasibility and potential benefits of using 3D printing technology to develop personalized and customized porous tantalum implants for complex spinal fusion procedures. Porous tantalum prostheses with excellent biomechanical properties can be manufactured through SEBM. Meanwhile, 3D printing technology and customized prostheses can effectively improve surgical efficiency and achieve satisfactory clinical outcomes in the treatment of complex cervical and thoracic fusion. Future research should further validate these findings and explore the broader application prospects of this innovative surgical technique.

## Data Availability

The original contributions presented in the study are included in the article/Supplementary Material, further inquiries can be directed to the corresponding author.
