# Supplementary material for: Personalized porous tantalum implants crafted via 3D printing: new horizons in complex cervical-thoracic spinal fusion
Source: Front Bioeng Biotechnol. 2025 Aug 21;13:1625650. doi: 10.3389/fbioe.2025.1625650 (PMC12408612; doi:10.3389/fbioe.2025.1625650)

**In Vitro Cytotoxicity Test Research**

A 3 cm² 3D-printed porous tantalum sample was incubated with 1 mL of serum-containing medium at 37°C under shaking for 24 hours, followed by filtration through a 0.22 μm membrane to collect the filtrate. The filtrate was then diluted at ratios of 100%, 75%, 50%, and 25% and subsequently exposed to mouse fibroblast cells (L929). Cells were seeded in a 96-well plate (100 μL/well) with seven experimental groups established:

1. Experimental groups: 100%, 75%, 50%, and 25% diluted filtrates (100 μL/well);
2. Blank control group: complete medium only (200 μL/well);
3. Negative control group: DMEM medium supplemented with 10% FBS (solvent control, 200 μL/well);
4. Positive control group: medium containing 0.1% Triton X-100.

Each group consisted of six replicate wells and was incubated in a 37°C, 5% CO₂ incubator for 24 hours, after which cell morphology was observed.

The L929 cell viability was assessed using the MTT assay to determine the cytotoxicity of the porous Ta. After discarding the medium, 20 μL of MTT solution (5 mg/mL) was added to each well. Following a 4-hour incubation at 37°C, the supernatant was removed, and 150 μL of DMSO was added per well. After shaking for 10 minutes, the absorbance at 490 nm (OD value) was measured using a microplate reader.

The in vitro cytotoxicity of the leachate from porous Ta was observed to assess its biosafety. Cell morphology revealed that in the blank control (Figure A) and the negative control (Figure B), discrete particles were present within the cytoplasm, with no evidence of cell lysis or a decline in cell proliferation. In the positive control group, the cell layer was almost completely destroyed (Table). After 24 hours of exposure, in the undiluted group, no more than 20% of the L929 cells exhibited shrinkage, loose adherence or morphological changes, with a slight inhibition of cell growth, indicating mild cytotoxicity (Figure C). In the dilution groups (75%/50%/25%), only discrete particles were observed within the cytoplasm, without cell lysis (Figure D/E/F).

In the MTT assay, we referred the blank control group as a 100% cell viability. After 24 hours of exposure, the cell viability in all concentration groups were greater than 75%, exceeding the 70% viability rate of the blank control group, indicating that the sample had no potential cytotoxicity (Table).

Table: Results of in vitro cytotoxicity test (MTT assay) (x ± s, n = 6)

| Group | OD_570_ | cell viability (%) |
| --- | --- | --- |
| Blank control | 1.094±0.101 | 100 |
| Negative control | 1.072±0.076 | 97.99 |
| Undiluted (100%) | 0.827±0.056 | 75.59 |
| 75% diluent | 0.985±0.086 | 90.04 |
| 50% diluent | 1.080±0.191 | 98.72 |
| 25% diluent | 1.082±0.185 | 98.90 |
| Positive control | 0.056±0.003 | 5.12 |

Figure: In vitro cellular morphological observations of porous tantalum fabricated via Selective Electron Beam Melting (SEBM) (Magnification: 100×). A) Blank control group; B) Negative control group; C) Undiluted (100% concentration); D) 75% dilution; E) 50% dilution; F) 25% dilution.


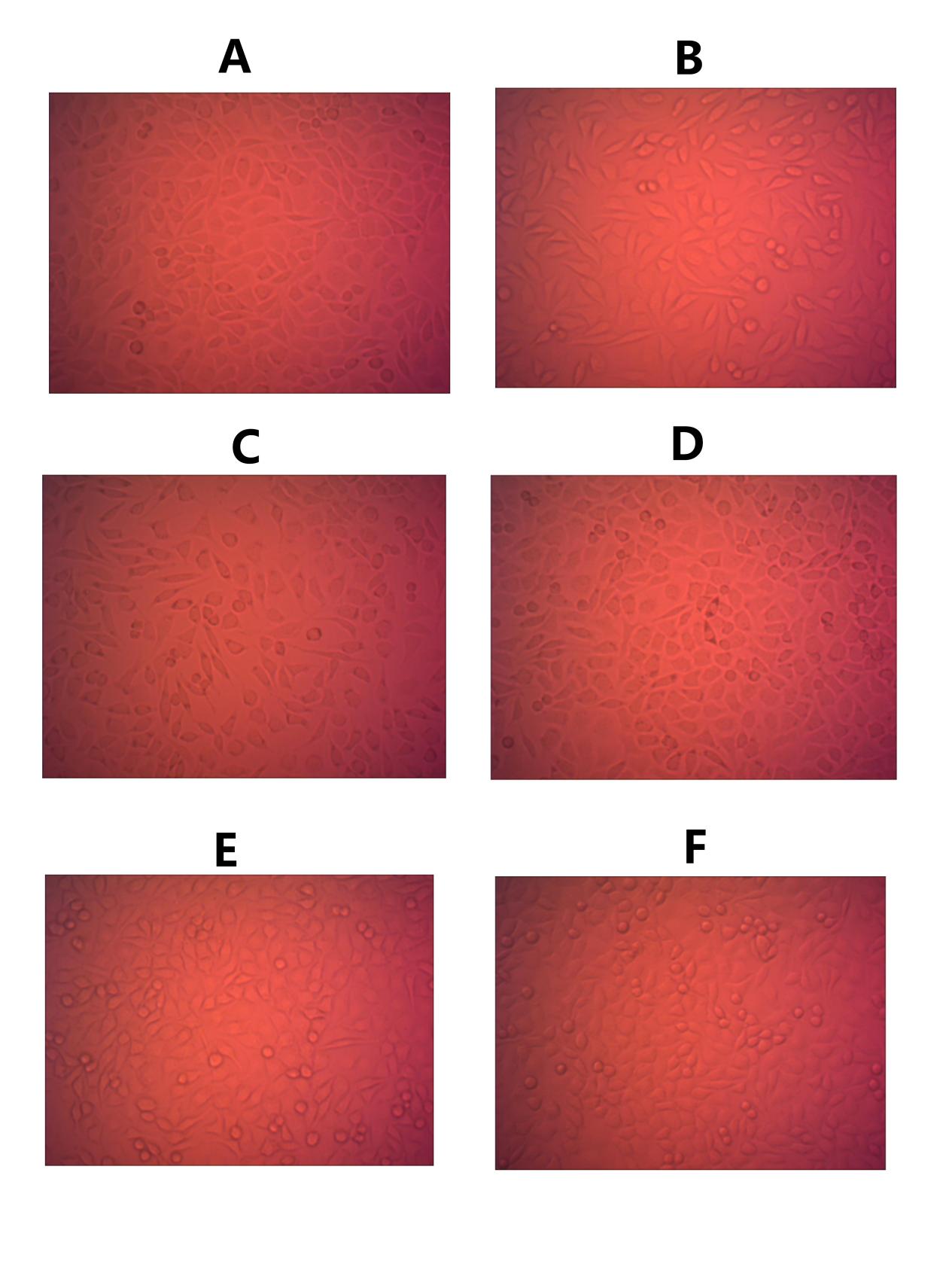

Supplement: Supplementary file 1 [file DataSheet1.docx]
